# Michael King, MD, PhD, FRCP, FRCGP, FRCPsych

**DOI:** 10.1192/bjb.2021.127

**Published:** 2022-06

**Authors:** Helen Killaspy

Formerly Professor of Psychiatry, University College London, and Director of the Division of Psychiatry, University College London, UK

**Figure:**
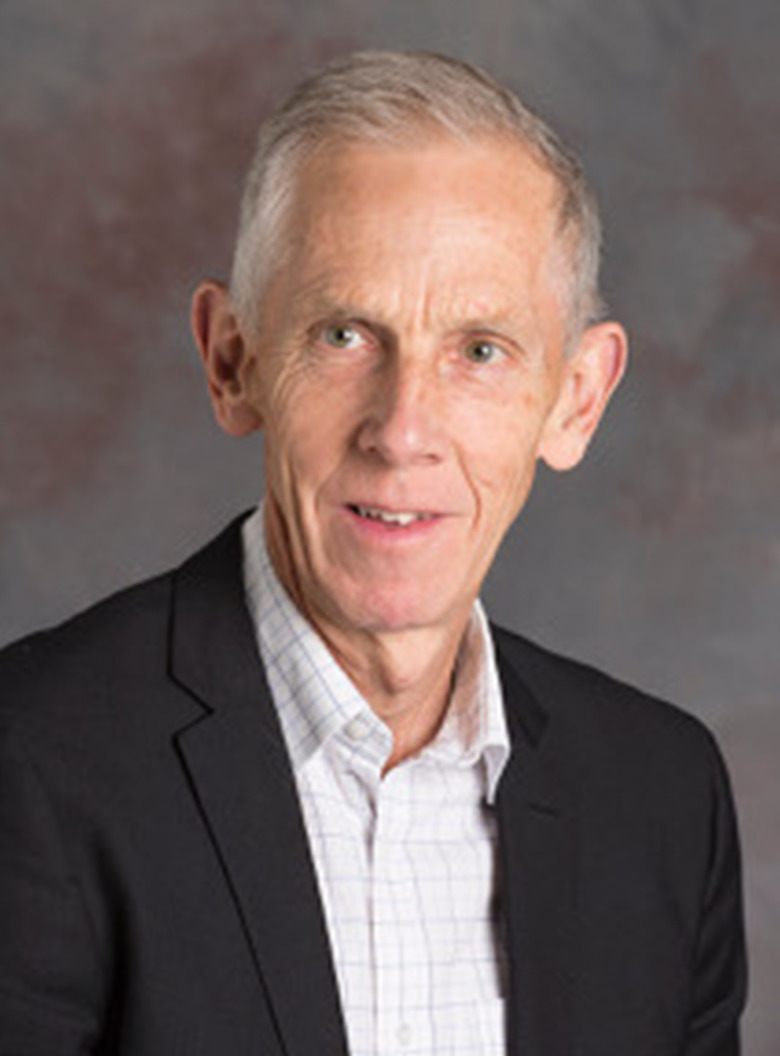

Professor Michael King, who died on 10 September 2021 aged 71, made numerous important contributions in primary care psychiatry, including risk prediction and the evaluation of complex interventions for mental disorders. However, his research interests went much further than this. Always curious and with an insatiable intellectual appetite, he was an academic polymath, often drawing on approaches from other fields and applying them to psychiatric research. He was particularly fascinated by subjects that were difficult to study, such as improving mental health at the end of life for people with cancer, the role of spiritual beliefs in mental well-being, and the mental health and stigma experienced by people from sexual minorities. Michael was a brilliant methodologist and a prolific researcher, producing almost 800 peer-reviewed publications, attracting over £45 million in grant income and supervising 30 PhD students over the course of his career. He was also tremendously generous and effective in supporting the development of junior clinical and non-clinical academics, many of whom are leaders in their fields today.

Michael believed that visibility was key in addressing homophobia in society and, as an out gay man, he was also a courageous advocate for the rights of LGBT people, drawing on the scientific evidence to make his case. In the 1990s he was instrumental in changing how the cause of death was recorded for victims of AIDS, to mitigate the associated stigma without compromising the collection of accurate statistics. His research into male victims of sexual violence influenced the current legal definition of male rape. He was often called as an expert witness in cases of child custody involving lesbian and gay parents. He also gave expert evidence to the Church of England Synod on same-sex marriage and the ordination of LGBT ministers. In 2001, with Annie Bartlett, he co-founded the Royal College of Psychiatrists’ LGBT special interest group (now the Rainbow SIG). Michael contributed hugely to the work of this group, presenting at numerous conferences and providing wise counsel to the College on relevant, often contentious, matters of policy. He was able to remain calm, even when seriously provoked, always presenting his arguments in an empathic, open and assured way. At the time of his death, he remained the foremost expert in LGB mental health in the UK and an international leader in this area. He also weathered a number of attacks on his reputation as a consequence of his courage in speaking up for sexual minorities. Although he was not someone who sought accolades or prizes, he was honoured to be invited to give the prestigious Beattie Smith lecture at the University of Melbourne in 2017, in recognition of his immense contribution to this field.

Michael was one of two brothers born in Christchurch, New Zealand on 10 February 1950, to Bruce, a farmer, and Patricia King. He completed his medical studies at the University of Auckland in 1976 before moving to the UK to train in family practice. In 1981 he began his training in psychiatry at the Maudsley Hospital, remaining firmly invested not just in psychiatry but in medicine and primary care throughout his career. He was awarded Membership and Fellowship of the medical Royal Colleges of all three specialties. He trained in psychiatric epidemiology at the General Practice Research Unit of the Institute of Psychiatry and gained both an MD (University of Auckland 1986) and PhD (University of London 1989) prior to his appointment as senior lecturer in the Department of Academic Psychiatry at the Royal Free Hospital School of Medicine in London. He became the Head of Department in 1995 while he was still only a Reader and, under his energetic and inspiring leadership, the Department rapidly expanded. Later, when the Medical School became part of University College London (UCL), he became Director of the UCL Division of Psychiatry, a role he retained until 2014.

As an adult, Michael learnt Spanish, French and German and developed a number of long-standing international collaborations, particularly in South America, Europe, India and Australasia. He was also an excellent clinician, setting up the psychosexual service at Camden and Islington NHS Foundation Trust for which he was the consultant psychiatrist for 30 years.

Michael met his life partner, Professor Irwin Nazareth, in 1984 at the Gay Medical Association. They celebrated their civil partnership in 2006 and married in 2017. In 2019 he contracted a rare non-tuberculous mycobacterium (NTM) infection, later found to be connected to the extremely rare lung condition pleuroparenchymal fibroelastosis, from which he died. As was typical of his approach to life, finding that no patient support group existed, he established one, NTM Patient Care UK (www.ntmpatientcare.uk). Michael is survived by Irwin, two nieces and a nephew. He is very much missed by his family and by his many friends and colleagues across the world.

